# Impact of Smoking on Women During the Covid-19 Pandemic

**DOI:** 10.3389/fmed.2021.584061

**Published:** 2021-04-30

**Authors:** Florin Dumitru Mihaltan, Armand-Gabriel Rajnoveanu, Ruxandra-Mioara Rajnoveanu

**Affiliations:** ^1^Pneumology Department, Marius Nasta National Institute of Pneumology, Bucharest, Romania; ^2^Occupational Medicine Department, Iuliu Hatieganu University of Medicine and Pharmacy, Cluj-Napoca, Romania; ^3^Pneumology Department, Iuliu Hatieganu University of Medicine and Pharmacy, Cluj-Napoca, Romania

**Keywords:** women, smoking, COVID-19, pandemic, SARS-CoV-2, gender, sex

## Abstract

The coronavirus disease 2019 (COVID-19) brought in 2020 an important challenge for health-care systems and authorities. Smoking and its influence on this disease remain, after months of the pandemic, one of the debatable risk factors. From the literature point of view, the focus of most articles is on smoking as a possible general risk factor for all analyzed populations. Women tend to represent a more significant population in exposed occupations. In our mini-review, we try to dig deeper, looking for gender-related health effects of smoking in this pandemic context, its effects on the infection with this novel severe acute respiratory syndrome coronavirus 2 (SARS-CoV-2), on illness severity, and on the rate of hospitalization and mortality. Despite the fact that the male gender is reported in many articles as a predictor of a poor outcome, we suggest that further research is needed to confirm or deny these relationships. Moreover, studies focusing specifically on women in these study populations are required.

## Introduction

The coronavirus disease 2019 (COVID-19) is a new global threat. It started in December 2019 in Wuhan, China, and the novel coronavirus was identified on January 7, 2020 in a patient with pneumonia and severe acute respiratory syndrome (SARS) (1). Smoking is considered a risk factor for COVID-19 patients, which increases the susceptibility to bacterial and viral infections in a multifactorial way (by the alteration of muco-ciliary escalator and immunological defense mechanisms) and enhances inflammation in a lung already affected by structural and immunological alterations (2). Tobacco consumption also increases the expression of angiotensin-converting enzyme 2 (ACE-2), which is a binding receptor for severe acute respiratory syndrome coronavirus 2 (SARS-CoV-2) (3). Some previous evidence related to smoking as a risk factor came from studies focused on the vulnerability of patients with smoking history to influenza and other coronavirus infections like the Middle East respiratory syndrome (MERS)-related coronavirus (4, 5). The focus on women who smoke is important and well-justified in reference to their professional profiles and roles as caregivers in some countries, which may make them more vulnerable to the impact of COVID-19.

We conducted a systematic mini-review and meta-analysis considering the association of smoking with SARS-CoV-2 infection or COVID-19 based on electronic search in MEDLINE (PubMed interface), Scopus, and Web of Science using the keywords “smoking” AND “COVID-19,” OR “2019-nCoV,” OR “SARS-CoV-2,” without language or time restriction. To collect maximum data, we did not limit the search to gender-related articles. The abstract and full text of all documents identified with these search criteria were reviewed to identify information on gender-related aspects of smoking and COVID-19.

### Smoking and COVID-19: An Active Debate

COVID-19 does not seem to affect patients uniformly. There are studies that found nearly similar infection rates in both genders (6), while others like the extensive meta-analysis on 221,195 patients published recently in BMJ Open, revealed a pooled prevalence of COVID-19 among men aged 55.00 years (range, 51.43–56.58, *p* < 0.001), with smoking and alcohol consumption being suggested as possible explanations (7). Another meta-analysis on 233 studies that looked at the impact of smoking reported some surprising results: current smokers appear to be at a reduced risk of SARS-CoV-2 infection compared to never smokers (8). Similar findings were published by Farsalinos et al. (9) through the analysis of the pooled prevalence of the current smoking status across 11 case series of Chinese patients. The current smoking status had a significantly lower-than-expected gender and age-adjusted prevalence in COVID-19 patients. Italian authors, without any comments on gender influences (10), also reported that 84.8% of Italian patients with COVID-19 pneumonia never smoked, and 15.2% were ex-smokers, in the Veneto region, with a prevalence of 22.4% smokers and 21.2% ex-smokers.

Looking more in-depth at smoking and COVID-19 relationship, a meta-analysis on 31,871 patients revealed a positive association between smoking and disease progression [29.2% in smokers and 21.1% in non-smokers with an odds ratio (OR): 1.56, 95% confidence interval (CI): 1.32–1.83, *p* = 0.001]. Moreover, the same research showed that smoking seems to be an independent risk factor for COVID-19 progression especially in younger people (11). These findings are confirmed by Spanish researchers (12) who concluded that current and past smoking are responsible for more severe forms of COVID-19, including more cases treated in intensive care units, intubations, and even death. Smoking was cited in the list of characteristics associated with severe SARS-CoV-2 infection (OR: 1.54, 95% CI 1.07–2.22), alongside cerebrovascular and cardiovascular disease, chronic obstructive pulmonary disease (COPD), diabetes, hypertension, and the male sex (13).

Apparently, in the US, there were 15.6% male and 12% female smokers in the general population in 2018 with a 1.3% prevalence for COVID-19 in current smoker patients (14). In China, there is a 1.4% prevalence of COVID-19 in current smoker patients vs. the prevalence of smoking in general population (52% in males and 2.5% in females) (15). However, there are many limitations to these studies, including small study populations, only inpatients as subjects, no age- and sex-matched control groups, or a high rate of missing data. Other limitations are generated by the percentage of asymptomatic patients. Furthermore, identifying asymptomatic infections is difficult, which makes it difficult to prevent and control this pandemic and understand the relationship of smoking habits in these populations (16). French studies sustaining the nicotinic hypothesis (17, 18) reported other interesting gender-related details that there are no statistical differences when it comes to comorbidities in inpatients and outpatients at different age groups. The only statistical gender-related differences are for diabetes, immunodeficiency, and hypertension. However, these studies are problematic because neither study is peer reviewed. The lead author of the first one has a deep and long-standing link to the tobacco industry and proposes a new hypothesis inconsistent with the broader emerging literature on the links between smoking and COVID-19 (19). The second study was based on results from one small hospital where patients were not representative of the larger population and excluded intensive care and asymptomatic patients (19).

These results that showed lower prevalence of smoking in COVID-19 patients compared to the general population suggested the protective role of nicotine. A possible explanation was formulated by Abdel Massih et al., which was based on the decreasing effect of smoking on cellular levels of furin, a cleavage enzyme increasingly recognized in the pathogenesis of metabolic syndrome, and found in high serum levels of obese and diabetic patients (20).

### Smoking, Gender, and Hospitalization for COVID-19

Authors of 27 observational studies found that smokers represented 1.4–18.5% of hospitalized adults with COVID-19 (14, 21, 22). In *The New England Journal of Medicine* article published by Guan et al. titled, “Clinical Characteristics of Coronavirus Disease 2019 in China” (23), it showed that compared to non-smokers, smokers are 2.4 times more likely to be admitted to an intensive care unit, require mechanical ventilation, or die, with no gender-related correlation being studied. Smoking (ex-smokers, in particular) was associated with greater risk of COVID-19 hospitalization (24).

In a United Kingdom study, the lifestyle characteristics impact on patients hospitalized for viral infection (0.2% of the whole study group) compared to an analytical population of 387,109 participants (56.2 ± 8.0 years; 55.1% women), found a risk ratio adjusted for age, gender, and mutually for each lifestyle factor (including smoking) of 1.42 (1.79 vs. 1.12) (25).

A New York City research on factors associated with hospital admission revealed the male sex (OR: 2.8, 95% CI: 2.4–3.2, average marginal effect 16%) as a predictor, alongside age, heart failure, and chronic kidney disease (26). Another interesting finding of this article was the lower risk for hospital admission of former or current smokers; however, unknown smoking status was associated with a higher risk, and this may indicate a major bias that may arise in these studies.

### Smoking, Gender, and COVID-19 Severity

The presence of comorbidities may play an important role in disease progression. They are associated with a substantially increased risk of severe prognosis (27). Chinese studies provide a strong evidence regarding the association that male COVID-19 patients have higher rates of comorbidities than women (15, 23, 28). These comorbidities with higher rates in Chinese men compared to women are as follows: type 2 diabetes mellitus, arterial hypertension, COPD, lung cancer, and other smoking-related diseases (27). Some authors estimated 6% [with uncertainty interval (UI): 3–12] of males to be at high risk of severe COVID-19 infections compared to 3% (UI: 2–7) of females (29). The prevalence of one or more comorbidities depends on age, with approximately 10% by the age of 25 years, 33% by the age of 50 years, and 66% by the age of 70 years, similar for both males and females (29). Compared to non-smokers, smokers who are hospitalized with COVID-19 have two to nine times higher risk of serious COVID-19 complications (30). This relationship is not evident for women. Studies that searched for correlations between different comorbidities and COVID-19 reported that most female cases with rheumatic diseases and COVID-19 were hospitalized, and patients were in the 50–65 years age group and were non-smokers (389 subjects, representing 75%) (31). Some authors that do not acknowledge the relationship between smoking and SARS-CoV-2 infection severity (32). Lippi et al. do not consider active smoking as a significant predictor of COVID-19 severity, based on a meta-analysis performed on data collected from four Chinese studies, but they admit the existence of an appreciable trend toward a higher risk of severe disease progression associated with active smoking. A strong evidence of smoking as a predictor for a severe outcome is revealed by a meta-analysis performed on 22 studies and 13,184 COVID-19 patients (33). Current smoking (OR: 1.98; 95% CI: 1.16–3.39) and former smoking (OR: 3.46; 95% CI: 2.46–4.85) were associated with severe diseases. Most of the included studies were completed in China (95%), and 55% of participants were males, but no data on gender statistics were reported. Similar results were published by Reddy et al. on patients with a smoking history, analyzing data of 47 eligible studies and 32,849 patients (34), but without gender-related data.

Although it is premature to assess all the aspects of serious and late complications of COVID-19, such as pulmonary fibrosis, some initial findings may be reported, with smoking as one of the possible predictors of progression (35).

### Smoking, Gender, and Mortality by COVID-19

Evidence from previous reports suggested a higher mortality by SARS and MERS in male patients. Smoking, genetic factors, and the impact of reproductive hormones on immune systems and inflammatory responses were investigated as implicated in this gender disparity (36). Regarding COVID-19, a significant positive relationship between death and smoking or former smoking was found by Williamson et al. They examined 17 million patient records to determine risk factors for 5,683 COVID-19 fatalities (37). This was valuable only when the statistical model was adjusted for age and sex. Mortality due to SARS-CoV-2 in China was higher among men (4.7%) than women (2.8%), which may also reflect the large sex differences in smoking habits in China (52.1% in men and 2.7% in women) (38, 39). For Western countries, although the prevalence of smoking in men were higher, the gender differences in mortality due to COVID-19 were not that high (40). Spanish authors reported similar rates of COVID-19 prevalence of 50.4 and 49.9% in men and women, respectively, but with a mortality rate of 4.7 and 2.6%, respectively. This was explained by gender-based differences, such as patterns and prevalence of smoking. In Spain, tobacco smoking prevalence in 2017 was 25.6% in men and 18.8% in women (41). It seems that mortality rates and severe outcomes are more frequent in males. Beside higher prevalence of smoking in men, another possible explanation might be the better personal hygiene in women, and sexual dimorphism. Women can also depend on the protecting estrogen immune-stimulating effect of the immune system (42). However, looking at the data of patients with COVID-19 from China, this explanation does not correlate with the distribution of severe cases (41.9% in women and 42.2% in men) (23).

### Gender-Related Smoking Particularities

Country particularities may partially explain the gender differences seen in a smoking habit. China is one of the countries with certain particularities in differences observed between male and female smoking prevalence, and also for COVID-19, male smokers seem to be more prevalent than females. This country may correlate COVID-19 epidemiological data with national studies already conducted in the previous years concerning smoking prevalence. In 1970, 68% of Chinese men were smokers (including those who had stopped due to health problems), and another 7% were ex-smokers (described as smokers that stopped by choice). In 1991, with a follow-up until December 31, 1999, there was another Chinese study published (43). There were fewer female smokers than male smokers, but there was a large intergenerational decrease in the female smoking prevalence, from about 10% prevalence in women born in the 1930s, to only about 1% in women born around the 1970s (44, 45). In the 2015 results, these differences were maintained; however, exposure to secondhand smoke had a prevalence of 27.7% in the general population (52.1% among men and 2.7% among women) (46). These differences may explain why female Chinese smokers seem to be less affected by COVID-19 than male smokers.

### Smoking-Cessation Programs in COVID-19 Pandemic Times

Is there any increased interest in smoking cessation during the first months of the COVID-19 pandemic? On the basis of different studies, it seems that there is no increase in the number of searches for smoking cessation (for both genders) on Google in the initial months of the COVID-19 pandemic. This could indicate that there has been no actual increase in smoking cessation during the pandemic (47). This is in contrast with the recommendations stipulated by Verall, according to whom, to optimize current health-cease (reduce) smoking, alongside the assurance of adequate hypertension and diabetes control and continue exercising, are in the frontline of commonly available and inexpensive immunomodulatory and anti-inflammatory medications for COVID-19 (48). Even though the role of vaping in virus susceptibility is yet to be determined, it is worth saying that vaping should not be seen as a safe alternative, and e-cigarettes in general have been shown to be of no help in smoking cessation (49). Another common problem for both genders, also affecting cessation desire, needs to be outlined. The tobacco industry uses subversive tactics to improve its image through corporate social responsibility activities (50, 51) covering subjects like the potential development of a tobacco-leaves-based vaccine against COVID-19 by British American Tobacco, studies claiming that nicotine has a protective effect against COVID-19 (17, 52). Even though we have no studies on smoking cessation in COVID-19 pandemic, trials from the surgical literature (53, 54) suggest that even 4 weeks of smoking cessation may decrease the risk of adverse outcomes and intubation. One of the recommendations of the World Health Organization, for smokers, in this period, is to quit this dependency (55). Stress and worsening of mental health, associated with this pandemic, are well-known predisposing factors for increase in smoking (quantity and frequency) as well as relapses (56). The COVID-19 pandemic disrupted ongoing clinical trials of smoking cessation and forced research teams to rapidly implement changes to assure access to counseling and monitoring by phone or internet communication tools (57, 58). Smoking cessation recovers airway ciliary clearance and immune function, being strongly encouraged as a public health measure to limit the global impact of COVID-19 (59).

## Discussion

Adopting simple lifestyle changes could lower the risk of severe forms of COVID-19. This is valid for both genders. We need clear messages for women concerning smoking, with well-defined strategies like those coming from English specialists: advice on how to avoid going back to increased smoking, to fight using fast-acting nicotine replacement products (nicotine gums, mouth sprays), or uptitrating on nicotine by dual use (combinations of short- and long-acting nicotine replacement therapy, and/or e-cigarettes) (60). These messages might also be targeted to some specific ethnic groups (black and south Asian people) and adapted for those with least advantaged socioeconomic status or educational level, as these groups seem to be more affected by this infection (61, 62).

There are currently no peer-reviewed studies published to assess the risk of SARS-CoV-2 infection among smokers in general, and in particular in women. Therefore, a well-designed population-based studies controlled for age, gender, and relevant underlying risk factors must be conducted. There are also several limitations of clinical protocols, including poor data quality and access to hospital data (63). More research is needed to clarify the relationship between smoking and COVID-19, and also a focus on the presence of women in these study populations. There is also a need for more evidence on the effect of passive smoking on the spread of SARS-CoV-2 infection. For both genders, future studies that test similarly large study populations reflecting the smoking habit rates described in the general population in COVID-19 patients are necessary. In this research, it is also important not to neglect and to identify asymptomatic cases and cases with mild symptoms that do not require hospital visits (64). The impact of smoking in women, during this COVID-19 pandemic, is not sufficiently studied.

## Author Contributions

FM: conceptualization, writing original draft, and review. A-GR and R-MR: resources, writing review, and editing. All authors contributed to the article and approved the submitted version.

## Conflict of Interest

The authors declare that the research was conducted in the absence of any commercial or financial relationships that could be construed as a potential conflict of interest.
